# The Strategic Prompting Scale (SPS) for Measuring Metacognitive Regulation in Human–AI Interaction

**DOI:** 10.3390/jintelligence14070141

**Published:** 2026-07-06

**Authors:** Rossella Suriano, Alessio Plebe

**Affiliations:** Department of Cognitive, Psychological and Pedagogical Sciences and Cultural Studies, University of Messina, 98100 Messina, Italy; aplebe@unime.it

**Keywords:** strategic prompting, metacognitive regulation, human–AI interaction, generative AI, self-regulated learning, psychometric evidence

## Abstract

The increasing use of Neural Language Models (NLMs) calls for strategic and metacognitive skills in prompt formulation, monitoring, and adaptation. This study presents the Strategic Prompting Scale (SPS), designed to measure the extent to which users apply planning, adaptation, and evaluation strategies during interactions with generative language models. After expert review and pilot testing, an exploratory factor analysis with 187 participants revealed a three-factor structure—Planning, Adaptation, and Evaluation—explaining 60.4% of the total variance. The final 15-item scale demonstrated good internal consistency. Confirmatory factor analysis with an independent sample of 406 participants supported the proposed factor structure. The SPS also showed theoretically expected associations with related constructs, including positive correlations with metacognition and critical thinking, and negative correlations with disengagement in AI interaction. Overall, the findings provide preliminary psychometric evidence for the SPS as a theory-driven instrument to assess metacognitive regulation and self-regulated behaviors in generative AI use, with potential applications in educational, professional, and research contexts.

## 1. Introduction

The growing adoption of Neural Language Models (NLMs) is profoundly transforming how students, professionals, and users in general search for, generate, and process content (Acciai et al., 2025; Federiakin et al., 2024; B. Wang et al., 2022). Interaction with these systems goes beyond a purely instrumental exchange: recent evidence suggests that NLMs can act as cognitive support, facilitating the progressive construction of meaning through a dialogue akin to that with an expert interlocutor (Akram et al., 2025; Huda et al., 2025; Liao et al., 2024; Zhou et al., 2025). From this perspective, effective interaction with NLMs requires users not only to provide instructions, but also to regulate their cognitive actions by planning goals, monitoring responses, adapting strategies, and evaluating the adequacy of generated outputs.

In this context, the concept of prompting assumes a central role. Prompting is defined as the process through which a user designs, formulates, and adjusts instructions provided to an NLM to guide the generation of outputs (Korzynski et al., 2023; Marvin et al., 2023; Tolzin et al., 2024; Walter, 2024). Recent studies highlight that the quality of prompts is critical for the effectiveness of AI-generated responses, emphasizing the need for not only technical but also strategic and reflective skills (Singh et al., 2025; Jacobsen & Weber, 2025). Crafting an effective prompt requires anticipating the desired type of response, structuring the request clearly, critically evaluating the output, and progressively refining the instructions based on feedback from the system (Davis & Lee, 2023; Knoth et al., 2024; Suriano et al., 2026). Thus, prompting can be conceptualized not merely as a technical procedure, but as a metacognitive activity through which users plan, monitor, regulate, and evaluate their interaction with AI systems.

In educational contexts, metacognitive prompting traditionally refers to cues or prompts that encourage students to reflect on their cognitive processes, monitor their learning, and self-regulate their study strategies (King, 1992; Schraw, 2001). Although this conceptualization was originally developed within student learning and educational research, the underlying metacognitive processes of planning, monitoring, regulation, and evaluation are not restricted to students. In the context of generative AI, these processes may also apply to broader groups of users, including academic staff, professionals, and general users who interact with NLMs for information seeking, content generation, problem solving, or decision support. Thus, in the present study, metacognitive prompting is used as a theoretical starting point for conceptualizing strategic prompting as a broader form of user self-regulation in human–AI interaction. More recently, related approaches have been extended to artificial intelligence systems, referring to strategies that prompt the model itself to reflect on the response-generation process, thereby improving the quality, accuracy, and usefulness of its outputs (Wei et al., 2022; Y. Wang & Zhao, 2024). However, strategic prompting differs from these approaches because it focuses on the user’s self-directed regulation of interaction with AI. Rather than examining prompts as external cues for students or as model-oriented reasoning techniques, strategic prompting concerns how users across different contexts plan, monitor, revise, and evaluate their own prompting behavior.

In the present study, we conceptualize Strategic Prompting as a context-specific manifestation of metacognitive regulation in human–AI interaction. Strategic Prompting refers to the extent to which users apply self-regulatory strategies—such as planning, monitoring, adapting, and evaluating prompts—to obtain coherent, relevant, and useful responses from generative language models. For example, a user may engage in strategic prompting by first clarifying the goal of the request, specifying the desired format or level of detail, and providing relevant contextual information before submitting a prompt. During the interaction, strategic prompting may involve monitoring whether the AI response is coherent and useful, adding examples or constraints when the output is too generic, and reformulating the prompt when the model does not adequately address the intended objective. After receiving the response, users may also evaluate its completeness, ask for clarification, request limitations or alternative perspectives, and revise the prompt accordingly. It can be understood as a continuum ranging from passive and reactive AI use, characterized by unplanned requests, limited adaptation, and minimal evaluation of outputs, to active and strategic AI use, in which users define the goal of the request, monitor response quality, and iteratively refine prompts based on feedback received. Accordingly, the present study introduces the Strategic Prompting Scale (SPS), a self-report instrument designed to assess planning, adaptation, and evaluation strategies during GenAI use.

### 1.1. Theoretical Framework

The concept of strategic prompting lies at the intersection of metacognition and self-regulation, applied specifically to interactions with neural language models (NLMs). It differs from educational metacognitive prompting—which provides external cues to guide users’ reflection on their cognitive processes—and from prompt engineering, which focuses on technical mastery of prompts, as it involves self-directed behavior aimed at actively regulating AI interaction. Strategic prompting is theoretically grounded in metacognition principles (Flavell, 1979; Schraw & Dennison, 1994), which describe the ability to monitor and regulate one’s thinking through processes such as planning, ongoing control, and evaluation of adopted strategies. This theoretical framework distinguishes between metacognitive knowledge—awareness of one’s cognitive processes, available strategies, and the conditions under which they are effective—and metacognitive regulation, which encompasses planning, monitoring, and revising operations that enable individuals to consciously guide task execution (Schraw & Dennison, 1994).

When strategic prompting is applied in educational or knowledge-acquisition contexts, it can be related to the framework of Self-Regulated Learning (SRL) (Zimmerman, 2002). In the SRL model, learning is conceived as a cyclical process in which individuals set goals, select strategies, monitor progress, and evaluate outcomes. Metacognitive components play a central role in coordinating planning, control, and review during task execution. From this perspective, strategic prompting can support the main phases of the self-regulatory cycle: (a) forethought—by promoting explicit goal-setting and initial planning; (b) performance—by facilitating monitoring and self-checking through reflective questioning; and (c) self-reflection—by prompting evaluation of response quality and potential revision of strategies.

However, it is crucial to conceptually distinguish strategic prompting from the SRL model. SRL represents an internal, endogenous, and cyclical process characterized by a relatively stable sequence of phases (forethought, performance, self-reflection). Strategic prompting, in contrast, constitutes an observable and actionable manifestation of users’ metacognitive and self-regulatory processes. It is flexible, non-sequential, and can be activated at any point during AI interaction. It does not reproduce the full self-regulatory cycle but can selectively trigger individual processes—such as planning, monitoring, or evaluation—in response to users’ contingent needs. In summary, strategic prompting represents an external, flexible, and selectively activatable mechanism that reflects and operationalizes metacognitive and self-regulatory processes without replacing them.

### 1.2. Current Measures and Limitations

Despite the rapid diffusion of generative artificial intelligence (AI) in educational and professional contexts, validated instruments capable of systematically measuring how users strategically regulate prompting during interactions with AI systems are still lacking. Traditional tools developed within the domains of metacognition and self-regulated learning have provided solid theoretical and methodological foundations for understanding students’ cognitive and metacognitive processes. Established scales such as the Motivated Strategies for Learning Questionnaire (MSLQ; Pintrich et al., 1991), the Metacognitive Awareness Inventory (MAI; Schraw & Dennison, 1994), the Self-Regulated Learning Perception Scale (SRLPS; Turan et al., 2009), and the Self-Regulated Knowledge Scale–University (SRKS-U; Manganelli et al., 2015) assess various aspects of learning regulation, including motivation, cognitive and metacognitive strategies, time management, planning, monitoring, and evaluation of one’s activities. However, while these instruments are robust, they were not designed to capture the dialogic, generative, and iterative processes that characterize interaction with generative AI systems. Unlike traditional search tools or static information sources, GenAI requires users to actively guide the production of responses through prompt formulation, monitoring, revision, adaptation, and evaluation. Therefore, existing instruments do not adequately assess the control, adaptation, and reflective strategies inherent to prompting.

With the expansion of online learning and the increasing use of AI in educational processes, more recent instruments have been developed to evaluate digital competencies and self-regulatory strategies in technology-mediated environments. For example, the Self-Regulated Learning for Online Learning (SRL-O; Tarchi et al., 2024) includes dimensions such as academic self-efficacy, intrinsic and extrinsic motivation, negative achievement-related emotions, planning and time management, metacognition, study environment organization, effort regulation, online social support, and operational strategies for digital work.

In parallel, instruments aimed at generative AI literacy have begun to emerge. The Generative AI Literacy for Learning Scale (GenAI-LLs; Gümüş & Kara, 2025) assesses learning needs analysis, prompt formulation and language skills, autonomous learning, and critical thinking. The Meta AI Literacy Scale (MAILS; Carolus et al., 2023) measures practical AI application, understanding of core concepts, the ability to recognize AI-based systems, ethical awareness, and the capacity to create AI-based applications, including transversal dimensions of self-efficacy and self-management. The ChatGPT Literacy Scale (Lee & Park, 2024), in contrast, focuses on technical, communicative, and ethical competencies, as well as critical evaluation and creative application of generative AI.

Although these instruments represent an important step toward measuring AI literacy, they present substantial limitations: they do not investigate the strategies through which users formulate, refine, monitor, and evaluate prompts, nor how metacognitive regulation is enacted during iterative interactions with generative models.

The only scale currently designed specifically to assess prompt engineering skills is the Prompt Engineering Competence Scale (PECS; Gibreel & Arpaci, 2025), which focuses on the technical structuring and adaptation of prompts. However, PECS emphasizes procedural and technical aspects, neglecting the strategic, reflective, and evaluative dimensions that characterize prompting in real-world human–AI interactions.

In summary, although instruments exist that assess general metacognition, self-regulated learning, digital literacy, and prompt engineering competencies, no scale integrates the strategic dimension of prompting. This highlights a clear theoretical and empirical gap and underscores the need to develop instruments capable of capturing the dynamic, iterative, and situated nature of strategic prompting in generative AI-mediated environments.

### 1.3. Objective and Contribution of the Proposed Scale

This study aims to develop the Strategic Prompting Scale (SPS) and provide preliminary psychometric evidence regarding its factorial structure, reliability, and theoretically expected associations with related and less-related constructs. The development of the SPS addresses the need for a tool capable of assessing the extent to which users engage in planning, monitoring, adaptation, and evaluation during interactions with artificial intelligence. While the scale does not directly label users as strategic or passive, higher scores indicate a more deliberate and self-regulated use of AI. The scale fills a gap in the literature, as previous studies have focused separately on general cognitive/metacognitive strategies, AI literacy, or operational prompt engineering skills, without integrating these dimensions into a single construct applicable to human–AI interaction. In doing so, the SPS offers both theoretical and practical contributions. Theoretically, it enhances understanding of how metacognitive regulation may be expressed during AI use. Practically, it provides a tool that can guide educational interventions and support research on strategic and reflective AI interaction.

## 2. Materials and Methods

### 2.1. Preliminary Phase: Item Generation, Expert Review, and Pilot Testing

Item generation followed a theory-driven deductive procedure. Because no validated instrument specifically assessing Strategic Prompting was available, the items were newly developed rather than directly adapted from an existing scale. First, the construct was defined on the basis of metacognitive regulation and self-regulated learning frameworks, with particular attention to planning, adaptation, and evaluation processes. These theoretical dimensions were then translated into observable behaviours during GenAI interaction, such as clarifying the goal of a prompt, providing contextual information, revising prompts after inadequate responses, and evaluating the coherence or limitations of AI-generated outputs. Items were included in the initial pool when they represented one of the three theoretical dimensions and referred specifically to prompt-based interaction with GenAI, rather than to general study strategies or broad self-regulatory behaviours. This process led to an initial pool of 18 items, reported in Table 1 together with the main supporting references.

The items were evaluated by a panel of three experts in psychometrics, educational psychology, and AI literacy. Each expert independently rated the items in terms of relevance, clarity, and representativeness using a 5-point Likert scale. Inter-rater agreement for each item was calculated using Cohen’s κ coefficient, yielding a mean value of κ = 0.86, indicating substantial agreement among the reviewers.

Items with low scores or ambiguous feedback were revised to improve conceptual precision and avoid overlap between dimensions. Specifically, the revisions mainly involved shortening some item formulations, replacing potentially ambiguous words with clearer terms, and simplifying sentence structure to improve readability. These changes did not alter the intended theoretical content of the items but aimed to make their wording clearer and more directly understandable for respondents. Subsequently, a pilot test was conducted with 48 participants to identify potential issues and assess each item’s ability to discriminate between individuals with high and low scores. After completing the items, participants were asked to provide qualitative feedback through open-ended questions, such as: “Were there any items that seemed unclear or ambiguous?” and “Can you explain in your own words what you intended to respond to this item?” Additionally, for each item, participants rated clarity on a five-point Likert scale (1 = not clear at all, 5 = very clear), allowing the identification of potential interpretation difficulties or ambiguities.

Item discrimination was assessed using item-rest correlation, defined as the correlation between each item and the sum of the remaining items in the scale. This indicator simultaneously evaluates internal consistency and the capacity to differentiate between participants with low and high scores on the scale. The obtained item-rest correlation values ranged from 0.423 to 0.658, indicating that all items contributed satisfactorily to the discrimination among respondents. Items with higher values, such as item 7 (r = 0.658), showed particularly effective discrimination, while even items with lower values (e.g., item 18, r = 0.423) remained acceptable according to psychometric standards (≥0.30). Since all items demonstrated adequate correlations, no item was removed, confirming the preliminary robustness of the 18-item pool for the SPS (Table 2).

No item was removed during the preliminary phase because all items showed acceptable content relevance after expert revision and adequate item-rest correlations in the pilot test. Item exclusion was therefore conducted only in the subsequent exploratory factor analysis, based on empirical criteria such as insufficient factor loadings or uninterpretable cross-loadings.

### 2.2. Study 1

#### 2.2.1. Sample

The sample consisted of 187 volunteer participants recruited in university educational settings through in-class announcements and email invitations. All participants provided written informed consent prior to completing the SPS online, administered anonymously via Google Forms. The gender distribution was balanced, with 97 females (51.9%) and 90 males (48.1%). Ages ranged from 18 to 30 years (M = 21.82, SD = 2.55). All participants were of Italian nationality; the majority resided in Southern Italy and the Islands (78.91%), 14.01% in Northern Italy, and 7.08% in Central Italy. Educational level was recorded as the highest qualification already completed: 90% of participants reported a high school diploma, 7.5% a bachelor’s degree, and 2.5% a master’s degree. Most participants were currently enrolled as university students (88.8%), while the remainder were employed (3.8%), self-employed (1.3%), unemployed (2.5%), or belonged to other professional categories. The reported fields of study referred to the university programmes in which student participants were enrolled or, for graduates, the field of their completed degree, and included psychology, medicine, law, and economics. Smartphone usage was high (M = 6.34 h/day, SD = 2.43). All participants reported using neural language models (NLMs); 95.6% specifically used ChatGPT, with a mean daily usage of 1.42 h (SD = 1.11; range 0–7). The value of 0 h indicated that some participants had experience with NLMs but did not report daily use at the time of data collection; in Study 1, this applied to two participants. These participants were retained because prior experience with NLMs was sufficient to complete the SPS, which assesses users’ self-reported strategies during GenAI interaction rather than the frequency of current daily use.

#### 2.2.2. Statistical Analyses

Data were analyzed using JASP 0.95.4.0. To investigate the underlying structure of the 18 items of the Strategic Prompting Scale (SPS), an exploratory factor analysis (EFA) was conducted. The number of factors to extract was determined by combining three criteria: Monte Carlo simulation-based Parallel Analysis, Kaiser’s criterion (eigenvalues greater than 1), and visual inspection of the Scree plot to identify the inflection point. Factor extraction was performed using Principal Axis Factoring, and oblique rotation (Oblimin) was applied to allow for potential correlations between factors. Internal consistency of the resulting scales was assessed using Cronbach’s alpha, with values ≥ 0.70 considered acceptable and values ≥ 0.80 considered good. The significance level for all analyses was set at α = 0.05.

#### 2.2.3. Results

The factorability of the correlation matrix was preliminarily assessed. The overall Kaiser-Meyer-Olkin (KMO) index was 0.854, falling within the “meritorious” range according to Kaiser and Rice’s (1974) guidelines. Measures of Sampling Adequacy (MSA) for individual items ranged from 0.750 to 0.921, well above the recommended threshold of 0.60. Bartlett’s test of sphericity was significant, χ^2^(153) = 1459.14, *p* < .001, rejecting the null hypothesis of an identity matrix. These indicators confirmed the suitability of the data for exploratory factor analysis (EFA).

**Figure 1:**
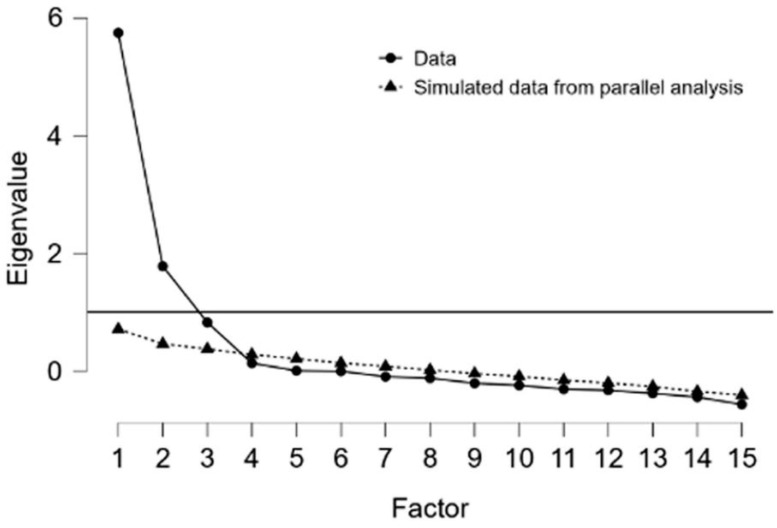
Scree plot of the observed eigenvalues and the Parallel Analysis.

To determine the optimal number of factors, multiple criteria were considered, including Monte Carlo simulation-based Parallel Analysis, the Kaiser criterion (eigenvalues > 1), and visual inspection of the Scree plot. The Scree plot showed a clear inflection point around the third factor, and Monte Carlo simulation-based Parallel Analysis indicated that the first three observed eigenvalues exceeded the corresponding simulated eigenvalues (Figure 1). Although the Kaiser criterion was inspected as an additional reference, the final decision to retain a three-factor solution was primarily based on Parallel Analysis, the Scree plot, and the theoretical interpretability of the solution. Overall, the three-factor solution was considered the most parsimonious and theoretically coherent representation of the data.

Factor extraction was performed using Principal Axis Factoring and, consistent with the expected correlation among factors, an oblique rotation (Oblimin) was applied. In the initial analysis of all 18 items, three items did not meet psychometric criteria: item 13 (“If I notice an unclear point in the AI response, I ask for clarification or revision on that specific point”) had insufficient loadings on all factors; item 8 (“If the AI’s response is unsatisfactory, I try to modify the prompt to make it clearer or more specific”) showed a very low loading; and item 5 (“I adapt the language or level of detail of the prompt according to the topic or purpose”) exhibited uninterpretable cross-loadings. These items were removed, resulting in a stable and interpretable 15-item solution.

The final three-factor solution was coherent with the Strategic Prompting model, delineating three distinct but correlated dimensions of metacognitive and self-regulated AI use. Together, the three factors explained approximately 60.4% of the total variance: Factor 1 accounted for 22.2%, Factor 2 for 19.7%, and Factor 3 for 18.6%.

Factor 1, Adaptation, reflects users’ ability to progressively modify and adapt the prompt based on AI feedback, through the addition of relevant details, targeted clarifications, and integration of new information. It represents monitoring and revision processes typical of metacognitive regulation.

Factor 2, Planning, involves defining the prompt’s objective, selecting necessary information, and intentionally structuring the request before submission, corresponding to anticipatory planning processes in metacognition.

Factor 3, Evaluation, focuses on the critical evaluation of the AI-generated output, including requesting clarifications, exploring potential limitations of the response, and self-checking comprehension, thus reflecting metacognitive evaluation and reflective processes.

Factor loadings for the final solution are presented in Table 3, while descriptive statistics, intercorrelations, and internal consistency indices for the three subscales are reported in Table 4. The three factors showed moderate correlations, consistent with the hypothesis of distinct yet interdependent constructs within the broader Strategic Prompting framework.

## 3. Study 2

The primary objective of Study 2 was to test the three-factor structure of the SPS in an independent sample and to provide preliminary evidence of construct validity through theoretically expected associations with related and less-related variables. The study aimed to test the previously identified three-factor structure, comprising Planning, Adaptation, and Evaluation, using a larger sample than that of Study 1.

To examine theoretically expected associations with related constructs, two conceptually related but item-independent measures were employed: the Metacognition in Creative Problem Solving Scale (MCPS; Urban & Urban, 2023) and the Critical Thinking Attitude Scale (CTAS; Fabio et al., 2025). The MCPS assesses general metacognitive skills applied to creative problem solving, including strategic planning, continuous monitoring, action regulation, and critical evaluation of outcomes, while the CTAS measures the disposition to analyze and reflectively evaluate one’s own decisions and knowledge.

The theoretical rationale for convergence is that Strategic Prompting involves conscious, self-regulated behavior aimed at optimizing interaction with a generative language model. Individuals who craft strategic prompts plan their requests, monitor the quality of responses, and adapt their instructions based on received feedback—all skills that directly reflect metacognitive processes and critical thinking abilities. Consequently, higher SPS scores are expected to be associated with higher levels of metacognition and critical thinking. Positive correlations with these measures would provide preliminary support for the interpretation of SPS scores as reflecting reflective and self-regulated behaviors consistent with metacognition theory.

To examine associations with a theoretically less adaptive pattern of AI interaction, a disengagement scale in interaction with ChatGPT, adapted from USEQ (Bernava et al., 2021) and widely used in previous studies (Gil-Gómez et al., 2017; Suriano et al., 2025) to measure boredom, low engagement, and reduced motivation in digital platform use, was employed. Since the SPS measures strategic and reflective behaviors, it is expected that more engaged and motivated individuals would exhibit higher scores, whereas disengaged individuals would not employ conscious prompting strategies. A negative correlation with disengagement would therefore be consistent with the interpretation that SPS scores reflect a construct distinct from passive or superficial attitudes in AI interaction.

### 3.1. Sample

The sample consisted of 406 volunteer participants, recruited as in Study 1. Gender distribution was balanced: 224 women (55.08%) and 182 men (44.92%). Ages ranged from 18 to 30 years (M = 22.07, SD = 2.69). All participants were Italian nationals; the majority resided in Southern Italy and the Islands (64.03%), 23.98% in the North, and 11.99% in the Center. Educational attainment was as follows: 79% held a high school diploma, 15% a bachelor’s degree, and 6% a master’s degree. Fields of study included psychology, medicine, law, and economics. Most participants were students (90.13%), with the remainder being employees (1.8%), self-employed (1.1%), unemployed (3.4%), or in other professional categories. Smartphone usage was high (M = 6.34 h/day, SD = 2.43). All participants reported using NLMs. A total of 97.1% reported using ChatGPT, with a mean daily usage of 1.29 h (SD = 1.13; range 0–5).

### 3.2. Measurement

The final version of the Strategic Prompting Scale (SPS) consists of 15 items distributed across three theoretically distinct factors: Planning, Adaptation, and Evaluation, each comprising 5 items. The SPS was administered in Italian to all participants. The English wording of the items is reported in the manuscript for presentation purposes, while the Italian version is provided in Appendix A. The items describe cognitive and behavioral strategies used by users when interacting with generative artificial intelligence systems. Participants responded using a 5-point Likert scale, ranging from 1 = ‘Not at all true for me’ to 5 = ‘Very true for me.’ The total score can range from 15 to 75, with lower scores indicating a less strategic approach to prompting and higher scores reflecting intensive and deliberate use of planning, adaptation, and evaluation strategies during AI interaction.

The Metacognition in Creative Problem-Solving Scale (MCPS), developed by Urban and Urban (2023), was used to assess individuals’ self-evaluation of their metacognitive abilities during creative problem-solving. The scale measures engagement in four key processes: planning, which involves the anticipatory definition of creative goals and strategies; monitoring, referring to the ongoing assessment of progress; regulation, which entails adapting strategies in response to challenges or new ideas; and evaluation, encompassing reflection on outcomes, including the originality of solutions. The MCPS consists of 11 items rated on a 7-point Likert scale ranging from 1 (Never) to 7 (Always), allowing for the computation of both a total score and subscores for each metacognitive process. In the present study, the scale demonstrated excellent internal consistency, with a Cronbach’s alpha of 0.86.

The Italian version of the Critical Thinking Attitude Scale (CTAS; Fabio et al., 2025) was used to assess participants’ inclination to engage in analytical, evaluative, and metacognitive reflection. The CTAS consists of 26 items measuring four subscales: Systematicity (nine items), Search for Truth and Openness (six items), Analyticity (four items), and Inquisitiveness (seven items). Participants responded on a Likert scale ranging from 1 = strongly disagree to 5 = strongly agree, with higher scores indicating a stronger disposition toward critical thinking. In the present study, the CTAS demonstrated good internal consistency, with Cronbach’s alpha values above 0.78 for all factors.

To assess the level of disengagement during the use of the NLM, a questionnaire adapted from the USEQ (Bernava et al., 2021; Gil-Gómez et al., 2017) was used. It consisted of 4 items aimed at investigating the degree of interest shown by users during interaction with the system. The disengagement measure included two directly worded items reflecting disengagement, such as “I feel bored while using the chatbot,” and two positively worded engagement items, such as “I feel engaged while using the chatbot,” which were reverse-coded. This scoring procedure ensured that higher total scores consistently reflected greater disengagement during interaction with the NLM. Participants responded on a 5-point Likert scale ranging from 1 = not at all to 5 = very much. Internal consistency analysis showed a Cronbach’s alpha of α = 0.83.

### 3.3. Statistical Analysis

Confirmatory factor analyses (CFA) were conducted using JASP 0.95.4.0 to test the three-factor structure of the SPS identified in Study 1. Analyses employed Maximum Likelihood (ML) estimation, appropriate for continuous and approximately normally distributed data. Model adequacy was evaluated using several fit indices, including the chi-square/degrees of freedom ratio (χ^2^/df), Comparative Fit Index (CFI), Goodness of Fit Index (GFI), and Root Mean Square Error of Approximation (RMSEA). To examine preliminary evidence of construct validity, Pearson correlations were computed between the SPS and the Metacognition in Creative Problem Solving Scale (MCPS; Urban & Urban, 2023), the Critical Thinking Attitude Scale (CTAS; Fabio et al., 2025), and a measure of disengagement in interaction with ChatGPT adapted from the USEQ (Bernava et al., 2021; Gil-Gómez et al., 2017). The significance level for all analyses was set at α = 0.05.

### 3.4. Results

The three-factor structure of the SPS identified in Study 1 was supported through CFA. Although the chi-square test was significant, χ^2^(87) = 172.40, *p* < .001, the relative chi-square indicated acceptable fit, χ^2^/df ≈ 1.98. Additional fit indices suggested good model adequacy: CFI = 0.975, TLI/NNFI = 0.969, NFI = 0.952, IFI = 0.975, RNI = 0.975, PNFI = 0.789, RFI = 0.943, GFI = 0.987, RMSEA = 0.049, 90% CI [0.038, 0.059], PCLOSE = 0.570, and SRMR = 0.046. Overall, these results support the proposed three-factor structure of the SPS.

Correlations between SPS factors and criterion variables are presented in Table 5, with all correlations significant at *p* < .01. The total SPS score showed moderate positive correlations with MCPS (r = 0.409) and CTAS (r = 0.241) and a negative correlation with Disengagement (r = −0.232). The Planning factor was positively associated with MCPS (r = 0.360) and CTAS (r = 0.226) and negatively correlated with Disengagement (r = −0.194). Similarly, the Adaptation factor showed positive correlations with MCPS (r = 0.262) and CTAS (r = 0.198) and a negative correlation with Disengagement (r = −0.186). Finally, the Evaluation factor displayed positive correlations with MCPS (r = 0.324) and CTAS (r = 0.148) and negative correlations with Disengagement (r = −0.163). Overall, these findings indicate that all SPS factors are positively associated with adaptive strategies, such as planning, adaptation, and creative thinking, and negatively correlated with disengagement behaviors, thereby providing preliminary support for the expected nomological pattern of associations.

The reliability of the three SPS factors and the total scale was assessed using ω (omega) and α (Cronbach’s alpha) coefficients. As reported in Table 6, ω coefficients ranged from 0.845 to 0.858 for the individual factors and reached 0.920 for the total scale, while α coefficients ranged from 0.812 to 0.847 for the factors and 0.867 for the total scale, indicating good internal consistency at both the factor and overall scale levels (Cronbach, 1951; McDonald, 1999). Internal convergence of the factors was evaluated using the Average Variance Extracted (AVE). AVE values for the three factors all exceeded 0.50 (Factor 1 = 0.610, Factor 2 = 0.625, Factor 3 = 0.542), suggesting that each factor explains an adequate proportion of the variance of its respective items and supporting the internal convergence of the three latent dimensions.

## 4. Discussion

The present study aimed to develop the Strategic Prompting Scale and provide preliminary psychometric evidence for its factorial structure, reliability, and theoretically expected associations. The results from the two studies provide preliminary support for the factorial structure and reliability of the SPS. Exploratory factor analysis in Study 1 revealed a three-dimensional structure consistent with the theoretical construct of Strategic Prompting, comprising Planning, Adaptation, and Evaluation, accounting for over 60% of the total variance. These findings suggest that user strategies during prompt formulation are not unitary behaviors but consist of distinct yet interrelated processes that reflect key components of metacognition and self-regulated learning (Flavell, 1979; Schraw & Dennison, 1994; Zimmerman, 2002, 2013).

The factorial structure was supported via confirmatory factor analysis in Study 2, and high internal consistency coefficients and AVE values above 0.50 support the psychometric adequacy of the scale. The expected pattern of associations was further supported by positive correlations between the SPS and measures of metacognition and critical thinking, and by negative correlations with disengagement during AI interaction. These results indicate that the SPS captures strategic and reflective behaviors, distinguishing them from passive or superficial attitudes in the use of generative systems.

The identification of three distinct dimensions has important theoretical implications. Planning highlights the importance of predefining goals and consciously structuring prompts, Adaptation reflects the ability to optimize behavior in response to model feedback, incorporating clarifications, additional details, and iterative revisions, and Evaluation indicates critical reflection of outputs, focusing on understanding, accuracy verification, and generalizability of generated responses. This multidimensional model reinforces the view that Strategic Prompting constitutes an observable manifestation of metacognitive processes, flexible and not constrained to the fixed sequences typical of self-regulated learning models.

### Strengths, Limitations, and Future Directions

The present study has several notable strengths. First, the SPS represents a novel psychometric instrument specifically designed to assess metacognitive and self-regulatory strategies in prompt formulation for generative language models. The scale integrates aspects of Planning, Adaptation, and Evaluation, providing a comprehensive measure of users’ strategic behaviors during AI interaction.

Despite these encouraging results, several limitations should be acknowledged. First, the sample mainly consisted of young Italian university students and was geographically concentrated in Southern Italy and the Islands, limiting the generalizability of the findings to other cultural, professional, or linguistic contexts. Second, the SPS relies exclusively on self-report measures, which may be influenced by social desirability bias or inaccurate perceptions of one’s own AI interaction strategies. Third, although the scale demonstrated a coherent factorial structure and good internal consistency, some items showed moderate loadings, suggesting that discriminative precision could be further optimized. Additionally, although most participants reported primarily using ChatGPT, the study was not restricted to a single generative AI system. The predominance of this model in the sample implies that the scale’s applicability to other language models or AI platforms remains to be examined. The present study did not explicitly assess whether participants interacted with GenAI systems through written or spoken prompts. Although the strategic processes assessed by the SPS may be relevant across written, spoken, and hybrid forms of interaction with GenAI systems, the present study did not explicitly distinguish between prompting modalities. Future research should therefore examine whether the scale functions similarly across text-based, voice-based, and multimodal prompting contexts. Fourth, some criterion measures used to examine theoretically expected associations were adapted or selected for their conceptual relevance rather than representing fully established Italian validation standards for the present context. Although their internal consistency was adequate, future studies should replicate these findings using independently validated Italian instruments and additional behavioral or performance-based indicators. Finally, the observed correlations between the SPS, metacognition, critical thinking, and disengagement do not allow for causal inferences. Although the findings are consistent with theoretical expectations, it cannot be concluded whether higher strategic prompting use leads to increased metacognition or vice versa.

Future research could expand and deepen the understanding of Strategic Prompting in several ways. First, it is advisable to test the SPS in more heterogeneous samples, including participants of different ages, cultural and professional backgrounds, and users of other languages or generative AI systems. Second, it would be useful to integrate the SPS with objective behavioral data, such as the analysis of authentic prompts, interaction log analysis, and prompt revision tracking, to compare perceived strategies with those actually employed during real human–AI interactions. Furthermore, future studies could investigate relationships between Strategic Prompting and concrete outcomes, such as the quality of AI-generated results, learning effectiveness, or professional productivity, using longitudinal or experimental designs to clarify directionality and causal mechanisms. Finally, the SPS can serve as a foundation for educational and training interventions aimed at developing planning, adaptation, and evaluation skills in conscious AI use, promoting more critical, reflective, and strategic users.

## Figures and Tables

**Table 1:** Items of the scale and main supporting references.

Item	Content	Sources
1. I adapt the formulation of my prompt to the specific objective I have in mind.	Planning	(Carolus et al., 2023; Gibreel & Arpaci, 2025; Lee & Park, 2024; Yurt, 2025)
2. I ensure that my prompt contains all the necessary information to obtain a useful response.	Planning	(Gibreel & Arpaci, 2025; Gümüş & Kara, 2025; Lee & Park, 2024)
3. Before writing a prompt, I try to clarify the goal of what I want to achieve with the AI.	Planning	(Pintrich et al., 1991; Schraw & Dennison, 1994; Turan et al., 2009)
4. When formulating a prompt, I include the necessary information so that the AI clearly understands the context.	Planning	(Gibreel & Arpaci, 2025; Lee & Park, 2024; Schraw & Dennison, 1994)
5. I adapt the language or level of detail of the prompt according to the topic or purpose.	Planning	(Gibreel & Arpaci, 2025; Gümüş & Kara, 2025; Lee & Park, 2024)
6. Before submitting a prompt, I often ask myself whether it is formulated clearly and precisely.	Planning	(Pintrich et al., 1991; Schraw & Dennison, 1994)
7. I integrate new information into the prompt to improve the quality of the response.	Adaptation	(Carolus et al., 2023; Gümüş & Kara, 2025; Lee & Park, 2024)
8. If the AI’s response is unsatisfactory, I try to modify the prompt to make it clearer or more specific.	Adaptation	(Carolus et al., 2023; Gümüş & Kara, 2025; Williamson, 2007)
9. When I do not get what I expect, I change strategy in formulating the prompt.	Adaptation	(Carolus et al., 2023; Gümüş & Kara, 2025; Turan et al., 2009)
10. If the AI’s response is generic, I add details or examples in the prompt to make it more specific.	Adaptation	(Carolus et al., 2023; Gümüş & Kara, 2025; Lee & Park, 2024)
11. I adapt the prompt when I notice that the AI has not correctly understood the objective of my request.	Adaptation	(Carolus et al., 2023; Gümüş & Kara, 2025; Williamson, 2007)
12. I progressively modify the prompt until the AI’s response exactly reflects what I am looking for.	Adaptation	(Carolus et al., 2023; Gümüş & Kara, 2025; Williamson, 2007)
13. If I notice an unclear point in the AI’s response, I ask for clarification or revision on that specific point.	Evaluation	(Carolus et al., 2023; Gümüş & Kara, 2025; Turan et al., 2009)
14. I rephrase in my own words what the AI has said and ask for confirmation to ensure I have understood correctly.	Evaluation	(Gümüş & Kara, 2025; Manganelli et al., 2015; Schraw & Dennison, 1994)
15. I ask whether the concept or information can be applied to other situations or contexts.	Evaluation	(Manganelli et al., 2015; Turan et al., 2009; Williamson, 2007)
16. I ask the AI to indicate any critical points, limitations, or ambiguities in the received response.	Evaluation	(Carolus et al., 2023; Gümüş & Kara, 2025; Lee & Park, 2024)
17. I ask the AI for an opinion or judgment on the response received to better evaluate its accuracy or completeness.	Evaluation	(Gümüş & Kara, 2025; Lee & Park, 2024; Williamson, 2007)
18. I ask the AI to explain the reasoning or process that led to its response, to verify its robustness.	Evaluation	(Gümüş & Kara, 2025; Lee & Park, 2024; Manganelli et al., 2015)

**Table 2:** Frequentist Individual Item Reliability Statistics.

Item	Item-Rest Correlation (r)	95% CI
1. I adapt the formulation of my prompt to the specific objective I have in mind.	0.539	0.420–0.640
2. I ensure that my prompt contains all the necessary information to obtain a useful response.	0.562	0.446–0.659
3. Before writing a prompt, I try to clarify the goal of what I want to achieve with the AI.	0.541	0.422–0.642
4. When formulating a prompt, I include the necessary information so that the AI clearly understands the context.	0.602	0.494–0.692
5. I adapt the language or level of detail of the prompt according to the topic or purpose.	0.615	0.509–0.703
6. Before submitting a prompt, I often ask myself whether it is formulated clearly and precisely.	0.545	0.427–0.645
7. I integrate new information into the prompt to improve the quality of the response.	0.658	0.561–0.737
8. If the AI’s response is unsatisfactory, I try to modify the prompt to make it clearer or more specific.	0.624	0.520–0.710
9. When I do not get what I expect, I change strategy in formulating the prompt.	0.551	0.434–0.650
10. If the AI’s response is generic, I add details or examples in the prompt to make it more specific.	0.636	0.534–0.719
11. I adapt the prompt when I notice that the AI has not correctly understood the objective of my request.	0.611	0.505–0.700
12. I progressively modify the prompt until the AI’s response exactly reflects what I am looking for.	0.528	0.407–0.671
13. If I notice an unclear point in the AI’s response, I ask for clarification or revision on that specific point.	0.630	0.527–0.715
14. I rephrase in my own words what the AI has said and ask for confirmation to ensure I have understood correctly.	0.463	0.333–0.576
15. I ask whether the concept or information can be applied to other situations or contexts.	0.450	0.318–0.565
16. I ask the AI to indicate any critical points, limitations, or ambiguities in the received response.	0.457	0.326–0.571
17. I ask the AI for an opinion or judgment on the response received to better evaluate its accuracy or completeness.	0.490	0.363–0.599
18. I ask the AI to explain the reasoning or process that led to its response, to verify its robustness.	0.423	0.288–0.542

**Table 3:** Factor loadings based on the EFA.

Item	F1	F2	F3
10. If the AI’s response is generic, I add details or examples in the prompt to make it more specific.	0.893		
12. I progressively modify the prompt until the AI’s response reflects exactly what I am looking for.	0.741		
11. I adapt the prompt when I notice that the AI has not correctly understood the objective of my request.	0.729		
9. When I do not obtain what I expect, I change strategy in formulating the prompt.	0.704		
7. I integrate new information into the prompt to improve the quality of the response.	0.680		
3. Before writing a prompt, I try to clarify the objective of what I want to obtain from the AI.		0.891	
4. When formulating a prompt, I include the necessary information so that the AI fully understands the context.		0.756	
2. I make sure my prompt contains all the necessary information to obtain a useful response.		0.748	
1. I adapt the wording of my prompt to the specific objective I have in mind.		0.581	
6. Before submitting a prompt, I often ask myself whether it is formulated clearly and precisely.		0.526	
17. I ask the AI for an opinion or judgment on the response received to better evaluate its accuracy or completeness.			0.840
16. I ask the AI to indicate any critical points, limitations, or ambiguities in the response received.			0.811
15. I ask whether the concept or information can be applied to other situations or contexts.			0.720
18. I ask the AI to explain the reasoning or process that led to its response, to verify its soundness.			0.703
14. I rephrase in my own words what the AI has said and ask for confirmation to ensure I have correctly understood it.			0.598

Note: Applied rotation method is oblimin.

**Table 4:** Descriptive statistics, correlations, and Cronbach’s alpha.

	M (SD)	α	1	2	3	4
1. SPS	56.77 (9.04)	0.87	-			
2. Planning	20.57 (3.32)	0.83	0.782 **	-		
3. Adaptation	20.99 (3.48)	0.85	0.776 **	0.594 **	-	
4. Evaluation	15.22 (4.77)	0.81	0.784 **	0.353 **	0.327 **	-

** *p* < .001.

**Table 5:** Correlations between SPS Factors and Criterion Variables.

	SPS	Planning	Adaptation	Evaluation
MCPS	0.409 **	0.360 **	0.262 **	0.324 **
CTAS	0.241 **	0.226 **	0.198 **	0.148 **
Disengagement	−0.232 **	−0.194 **	−0.186 **	−0.163 **

** *p* < .001.

**Table 6:** SPS Reliability Coefficients.

Factor	ω	α
1	0.858	0.827
2	0.845	0.847
3	0.846	0.812
Total	0.920	0.867

## Data Availability

The data are available upon request.

## Appendix A

**Table A1:** Italian version of SPS.

**Item**
1. Adatto la formulazione del mio prompt all’obiettivo specifico che ho in mente.
2. Mi assicuro che il mio prompt contenga tutte le informazioni necessarie per ottenere una risposta utile.
3. Prima di scrivere un prompt, cerco di chiarirmi l’obiettivo di ciò che voglio ottenere dall’AI.
4. Quando formulo un prompt includo le informazioni necessarie affinché l’AI comprenda bene il contesto.
6. Prima di inviare un prompt, spesso mi chiedo se è formulato in modo chiaro e preciso.
7. Integro nuove informazioni nel prompt per migliorare la qualità della risposta.
9. Quando non ottengo ciò che mi aspetto, cambio strategia nella formulazione del prompt.
10. Se la risposta dell’AI è generica, aggiungo dettagli o esempi nel prompt per renderla più specifica.
11. Adatto il prompt quando mi accorgo che l’AI non ha compreso correttamente l’obiettivo della mia richiesta.
12. Modifico progressivamente il prompt finché la risposta dell’AI non riflette esattamente ciò che sto cercando.
14. Riformulo con parole mie quanto l’AI ha detto e chiedo conferma per assicurarmi di aver capito correttamente.
15. Chiedo se il concetto o l’informazione può essere applicato a altre situazioni o contesti.
16. Chiedo all’AI di indicare eventuali punti critici, limiti o ambiguità nella risposta ricevuta.
17. Chiedo all’AI un parere o un giudizio sulla risposta ricevuta per valutarne meglio la sua correttezza o completezza.
18. Chiedo all’AI di spiegare il ragionamento o il processo che ha portato alla sua risposta, per verificarne la solidità.
